# Functional and Structural Characterization of a Eurytolerant Calsequestrin from the Intertidal Teleost *Fundulus heteroclitus*

**DOI:** 10.1371/journal.pone.0050801

**Published:** 2012-11-30

**Authors:** A. Carl Whittington, Tatyana E. Nienow, Christi L. Whittington, Timothy J. Fort, Theresa J. Grove

**Affiliations:** 1 Department of Chemistry and Biochemistry, Florida State University, Tallahassee, Florida, United States of America; 2 Department of Biology, Valdosta State University, Valdosta, Georgia, United States of America; 3 Department of Chemistry, University of South Florida, Tampa, Florida, United States of America; University of Sydney, Australia

## Abstract

Calsequestrins (CSQ) are high capacity, medium affinity, calcium-binding proteins present in the sarcoplasmic reticulum (SR) of cardiac and skeletal muscles. CSQ sequesters Ca^2+^ during muscle relaxation and increases the Ca^2+^-storage capacity of the SR. Mammalian CSQ has been well studied as a model of human disease, but little is known about the environmental adaptation of CSQ isoforms from poikilothermic organisms. The mummichog, *Fundulus heteroclitus*, is an intertidal fish that experiences significant daily and seasonal environmental fluctuations and is an interesting study system for investigations of adaptation at the protein level. We determined the full-length coding sequence of a CSQ isoform from skeletal muscle of *F. heteroclitus* (FCSQ) and characterized the function and structure of this CSQ. The dissociation constant (K_d_) of FCSQ is relatively insensitive to changes in temperature and pH, thus indicating that FCSQ is a eurytolerant protein. We identified and characterized a highly conserved salt bridge network in FCSQ that stabilizes the formation of front-to-front dimers, a process critical to CSQ function. The functional profile of FCSQ correlates with the natural history of *F. heteroclitus* suggesting that the eurytolerant function of FCSQ may be adaptive.

## Introduction

Fluctuating thermal environments pose a significant challenge to poikilothermic organisms. Animals that can exploit these variable thermal habitats are termed eurythermal. The eurythermal mummichog, *Fundulus heteroclitus* (Teleostei; Cyprinodontiformes), is a common killifish found in intertidal zones along the eastern coast of North America where it encounters fluctuating environmental, and thus physiological, temperatures during both seasonal and daily tidal cycles. Additionally, along its distribution, populations of *F. heteroclitus* experience an extreme thermal gradient: for every one degree change in latitude the mean annual water temperature changes by 1°C [1], [2]. Changes in temperature affect all biological and chemical processes, including the functions of and interactions between proteins [3]. The non-catalytic Ca^2+^-binding protein calsequestrin (CSQ), the focus of this study, undergoes large scale structural changes including folding and polymerization during Ca^2+^-binding [4] making it an interesting study system for investigations of environmental effects on protein structure and function.

Contraction and relaxation in skeletal and cardiac muscles are mediated in large part by the release and uptake of Ca^2+^ by the sarcoplasmic reticulum (SR). In vertebrates, CSQ is found in the SR where it is a high capacity Ca^2+^-binding protein localized to the terminal cisternae [5]–[8]. In mammals, CSQ sequesters a relatively large amount of Ca^2+^ (40–50 moles Ca^2+^ per mole CSQ) [5], [6], keeping levels of free Ca^2+^ low (0.2 mM–0.5 mM) within the SR during relaxation [9], [10]. Royer and Ríos [11] calculated that the total concentration of Ca^2+^ in the terminal cisternae of SR is between 35 mM and 175 mM. During a single twitch contraction there is a relatively small (∼10%) decrease in free Ca^2+^ in the SR, but more Ca^2+^ is released from the SR during repetitive contraction or tetanic events [5], [6], [9], [10], [12], [13]. In vertebrates, Ca^2+^ sequestration by CSQ enables fast twitch glycolytic and cardiac muscles to undergo repetitive high frequency cycles of contraction and relaxation without a cycle-by-cycle depression in developed tension, a key characteristic of both of these muscle types [14]–[18]. Additionally, CSQ may play a regulatory role in Ca^2+^ release through interactions with the ryanodine receptor (RyR) mediated through junctional SR proteins triadin and junctin [19], [20].

CSQ isoforms consist of three, contiguous thioredoxin-like domains each composed of a hydrophobic core surrounded by acidic residues that form a negatively charged surface [4], [21]. A model that is based on X-ray crystallographic evidence from mammalian cardiac and skeletal isoforms of CSQ illustrates the Ca^2+^-dependent conformational changes at the secondary, tertiary, and quaternary levels [4], [21], [22]. In the absence of Ca^2+^, CSQ has a random coil structure. As Ca^2+^ concentration increases to 10 µM, CSQ gains α-helical content as the three thioredoxin-like domains fold. Above 10 µM Ca^2+^, the full monomeric structure is reached. Front-to-front followed by back-to-back dimers form at 10 µM–1 mM Ca^2+^. Higher order linear polymers form above 1 mM Ca^2+^ [22], [23]. Back-to-back and front-to-front dimers form electronegative pockets where Ca^2+^ ions are transiently coordinated by pairs of negatively charged residues [12]. This transient binding may also allow faster diffusion of Ca^2+^ along the CSQ polymer and more effective delivery of Ca^2+^ to the RyR [11].

A number of non-covalent interactions among amino acid residues within mammalian CSQ have been identified that may be required for proper monomer folding, polymerization, and interaction with other proteins [4], [21]. Beard et al. [12] summarized the current understanding of CSQ polymerization and the role of the previously identified non-covalent contacts in polymerization. Interactions between the highly acidic C-terminal tails of two CSQ molecules lead to the formation of a back-to-back dimer, and a cluster of salt bridge interactions (Glu215-Lys86, Glu25-Lys24 and Glu169-Lys85) tethers this back-to-back formation. Front-to-front dimerization is stabilized by the salt bridge Glu55-Lys49, and similar to the back-to-back dimer, an acidic pocket forms between the monomers upon dimerization [12].

Paralogous isoforms of CSQ are expressed in vertebrate fast twitch skeletal muscle (CSQ1) and slow twitch skeletal and cardiac muscles (CSQ2). While a significant amount of research has focused on CSQ structure and function in mammals, much less is known about CSQ in fishes. CSQ has been identified in skeletal muscle and heater cells of fishes including carp (*Cyprinus carpio*) and blue marlin (*Makaira nigricans*), respectively [24], [25], but the CSQ isoform was not determined. Additionally, the sole (*Solea senegalensis*) expresses two of each CSQ isoform (e.g. CSQ2A and CSQ2B) [26]. In the present study, we determined the full-length coding sequence of a CSQ isoform from *F. heteroclitus* skeletal muscle (FCSQ), and subsequently expressed, purified, and characterized the function and structure of this CSQ. Interestingly, FCSQ is relatively insensitive to changes in temperature and pH, which indicates FCSQ is a eurytolerant protein [27]. The function of FCSQ correlates with the natural history of *F. heteroclitus;* proteins that are relatively insensitive to changes in environmental and physiological conditions may be advantageous to organisms that live in dynamic environments. Additionally, computational modeling of FCSQ reveals a highly conserved, charged interaction network that appears to be critical for proper CSQ function. This interaction network is equivalent to one identified in mammalian CSQ2 that is required for proper polymerization and Ca^2+^-binding [28]. The high level of conservation at the primary, secondary, and tertiary structural levels across CSQ isoforms and species indicates that conclusions based on studies of FCSQ will be broadly applicable to isoforms from a variety of vertebrates.

## Materials and Methods

### Ethical Statement

Animal Use Protocols (00014-2008 and 00038-2011) were approved by Valdosta State University’s Institutional Animal Care and Use Committee (Animal Welfare Assurance Number A4578-01). Care was taken to minimize pain and discomfort of animals.

### Collection of Specimens

*Fundulus heteroclitus* were caught off the coast of Jekyll Island, Georgia, USA (31°2′27.37′′N and 81°25′21.66′′W) with minnow traps. Fish were transported to Valdosta State University and held in re-circulating seawater (28 ppt) tanks until sacrificed. They were fed daily with flake food. Fish were euthanized with Finquel (MS-222) followed by spinal transection and pithing. Following removal of the skin, glycolytic skeletal muscle samples were taken from the axial musculature on the dorsal side of each individual immediately below or anterior to the dorsal fin. Muscle was either immediately used for RNA isolation or frozen at −80°C until use.

### RNA Isolation, cDNA Preparation, and Sequencing

Total RNA from *F. heteroclitus* was isolated from skeletal muscle using TRIzol reagent (Invitrogen) following manufacturer’s instructions. Single-strand cDNA was made from total RNA (5 µg) using oligo (dT) primer (Table 1) and Superscript III (Invitrogen) as per manufacturer’s protocol. cDNA was stored at −20°C until used to amplify *F. heteroclitus* CSQ cDNA (FCSQ).

**Table 1:** Primers used for amplification of *Fundulus heteroclitus* CSQ cDNA.

aPrimer Name	bPrimer Sequence	cDNA positions
RT-PCR Primer		
Oligo (dT)	5′-GCTTTTTTTTTTTTTTTTTTTT-3′	Poly(A) tail
Degenerate primers		
CSQ8F	5′-GCWGCYCARGTCTTGGARGA-3′	241 → 260
CSQ11R	5′-CCWGAMAGRACATCYTCKATCCA-3′	1084 ← 1106
*Fundulus* specific primers		
CSQAF	5′-GAGGAGCCAGTGGAGGTCATTG-3′	430 → 451
CSQBR	5′-AGATGATGCTGAGGTCAGGAAGG-3′	895 ← 917
CSQF1	5′-ACATTTCTCCATCCCTTCCAAG-3′	−112 → −91
CSQR1	5′-ATTTCATTTTGACCCCACCA-3′	1505 ← 1524
CSQF2	5′-ATGGAAAAGGGCCTGGAG-3′	58 → 76
CSQR2	3′-TTACTCATCATCATCATCATCATCA-3′	1280 ←1305

a F indicates forward primers; R indicates reverse primers.

b M = A or C; R = A or G; Y = C or T; W = A or T; K = G or T.

c Locations of primers are based on cDNA consensus sequence of *Fundulus heteroclitus* CSQ. Position 1 indicates the putative start site (ATG). Negative positions indicate positions upstream of the cDNA start site.

Initial amplifications of partial FCSQ single-strand cDNA were performed in 50 µl reaction volumes containing 100 ng/µl cDNA, 1X Advantage 2 polymerase buffer, 1X Advantage 2 polymerase mix (Clontech), 200 µM dNTPs, and 500 nM degenerate primers (Table 1). Degenerate primers, CSQ8F and CSQ11R, were designed from an alignment of available teleost CSQ1 and CSQ2 sequences (NCBI Accession Numbers: *Danio rerio* (zebrafish) CSQ2- NM_001002682; *F. heteroclitus* (mummichog) CSQ2- CV824367; *Takifugu rubripes* (pufferfish) CSQ1- CK829454 and CK829229. Reactions were as follows: initial denaturation of 2 min at 94°C, followed by 35 cycles of 45 sec at 94°C with an annealing step of 1 min at 62°C and an elongation step of 45 sec at 72°C; the reaction concluded with a final 5 min elongation at 72°C. Purified PCR products were ligated in pCR 2.1 TOPO vector, and TOP10 One Shot cells (Invitrogen) were transformed as per manufacturer’s instructions. Plasmids containing inserts were sequenced at the Florida State University Sequencing Facility.

5′ and 3′ RACE-ready cDNAs from FCSQ were prepared using the SMARTer RACE Amplification Kit (Clontech) following the manufacturer’s instructions. Based on partial FCSQ nucleotide sequences, gene specific primers CSQAF and CSQBR (Table 1) were designed for use in 3′ and 5′ RACE-PCR, respectively. Amplification was performed with Advantage 2 DNA polymerase with the gene specific primer and Universal primer. The 5′ and 3′ cDNA samples were prepared under the following conditions in 50 µl reactions: 2.5 µl first-strand cDNA, 1X Universal Primer Mix (UPM), 200 µM gene specific primer, 1X Advantage 2 polymerase buffer, 200 µM dNTPs, 1X Advantage 2 DNA polymerase mix. Touchdown PCR was performed as follows: 5 cycles of denaturation at 94°C for 30 sec and annealing and extension at 72°C for 3 min; 5 cycles of denaturation at 94°C for 30 sec, annealing at 70°C for 30 sec, and extension at 72°C for 3 min; and 25 cycles of denaturation at 94°C for 30 sec, annealing at 68°C for 30 sec, and extension at 72°C for 3 min. Samples were cloned, sequenced, and aligned as described above. The full-length coding sequence of FCSQ cDNA was then amplified with gene specific primers, CSQF1 and CSQR1 (Table 1). Reaction mixtures were identical to those used to obtain partial FCSQ sequence, and PCR reactions were similar except the extension time for each cycle was 75 sec. PCR products were cloned and sequenced as described above. A consensus sequence of FCSQ was generated from an alignment of full-length FCSQ from three individuals.

### Sequence Comparisons

To visualize the evolutionary relationship of FCSQ to other teleost CSQ isoforms, a phylogenetic tree was constructed. A multiple sequence alignment of teleost CSQ amino acid sequences including the SR targeting sequence (NCBI Accession Numbers: *Solea senegalensis* (sole) CSQ1A- BAG49512.1, CSQ1B- BAH85793.1, CSQ2A- BAG49513.1, CSQ2B- BAH85794.1; *Oreochromis niloticus* (tilapia) CSQ1A- XP_003457488.1, CSQ1B- XP_003454795.1, CSQ2A- XP_003455497.1, CSQ2B- XP_003440974.1; *Danio rerio* (zebrafish) CSQ1A- NP_001003620.2, CSQ1B- NP_001070192.1, CSQ2A- AAH75775.1; *Oncorhynchus mykiss* (trout) CSQ2A- NP_001153971.1; *Salmo salar* (salmon) CSQ1A- ACH70680.1) was performed using ClustalW on the EBI server with default parameters [29]. The phylogenetic tree was estimated from the sequence alignment using the neighbor-joining method in ClustalW with default parameters.

### Expression and Purification of Recombinant Teleost FCSQ

Full-length coding sequence of FCSQ minus the predicted signal sequence was amplified via PCR as described above using the gene specific primers CSQF2 and CSQR2 (Table 1). PCR products of expected length were ligated into PetBlue-1 AccepTor Vector (Novagen). Colonies containing plasmid with correct insert orientation were identified by digestion with XmnI, and the sequence was confirmed by sequencing. Tuner(DE3)pLacI cells (Novagen) were transformed with plasmid containing FCSQ as per manufacturer’s instructions. LB broth containing 100 µg/ml ampicillin and 1% glucose was inoculated and grown at 37°C with agitation until OD_600_ of 1. Temperature of the cultures was decreased to 15°C prior to induction of expression with 500 µM IPTG for 24 hours. Cells were harvested by centrifugation and stored at −80°C until FCSQ purification.

FCSQ was purified following the protocol described in Cala and Jones [30] with minor modifications. Briefly, cells were resuspended in Buffer A (10 mM MOPS, 0.1 mM EGTA, 500 mM NaCl, 1 mM DTT, pH 7.0) and lysed by intermittent sonication for 15–20 sec with cooling on ice after each sonication. The homogenate was centrifuged for 10 min at 14,000×g, and the supernatant was applied to a phenyl sepharose column (2×13 cm) equilibrated with Buffer A. The column was washed with 100 mL Buffer A. Protein was eluted with 100 mL Buffer A containing 10 mM CaCl_2_ followed by 100 mL 10 mM MOPS, pH 7.0. Fractions containing recombinant FCSQ were identified using 10% Tris/Glycine SDS-PAGE; gels were stained with Coomassie Blue. Fractions containing partially purified FCSQ were combined, exhaustively dialyzed against 0.1 M potassium phosphate, 1 mM EGTA, pH 7.1, and applied to a DEAE sepharose column (2.5×8 cm) equilibrated with 0.1 M potassium phosphate, 1 mM EGTA, pH 7.1. The column was washed with the same buffer, and FCSQ was eluted using a linear gradient of 0–1.0 M NaCl in 0.1 M potassium phosphate, 1 mM EGTA, pH 7.1.

Fractions containing FCSQ were identified using 10% Tris/Glycine SDS-PAGE as described above and combined; purified FCSQ was concentrated using Amicon Ultra-15 centrifugal filter devices with a 10 kDa cutoff (Millipore). FCSQ identity and purity were verified by 10% Tris/Glycine SDS-PAGE and subsequent staining with either Stains-all, which stains Ca^2+^-binding proteins blue [31], [32], or Coomassie Blue. In addition, N-terminal Edman sequencing of purified recombinant FCSQ was conducted at Florida State University’s Analytical Lab and Research Facility.

### Decalcification of FCSQ and Buffer Solutions

Divalent cations were removed from recombinant FCSQ by dialysis against 10 mM Tris-HCl, pH 7.5, containing 5% Chelex (w/v) at 4°C for 4 days with continuous stirring, changing buffer every 24 hours. Divalent cations were removed from buffer used in the fluorometric assay by the addition of 5% Chelex with continuous stirring at 4°C for 4 days, changing Chelex every 24 hours. Because decalcification with Chelex increases pH of buffers, the pH was adjusted immediately prior to assay using 1.0 N and 0.5 N HCl in 10 mM Tris-HCl that had been decalcified with 5% Chelex. Decalcification of FCSQ and buffers was confirmed by atomic absorption spectroscopy at Valdosta State University’s Advanced Spectroscopy and Biotechnology Facility.

### Intrinsic Fluorescence Assay and Calculation of K_d_

Concentration of decalcified FCSQ was determined using the bicinchoninic acid (BCA) protein assay kit (Sigma) prior to fluorescence assays. An intrinsic fluorescence assay that relies on tryptophan fluorescence was modified from Park et al. [23] and Hidalgo et al. [33]. For the temperature dependence experiments, CaCl_2_ standard (0.1 M; Orion) was added stepwise to FCSQ (15 µg/ml) in 10 mM Tris-HCl, pH 7.5, at 25°C. The pH was allowed to vary with temperature of the assay (10, 15, 25, or 35°C). For the pH dependence experiments, the pH of 10 mM Tris-HCl was adjusted to pH 6.6, 6.8, 7.2, or 7.5 at 25°C, and the assays were run at 15°C. The sample was allowed to equilibrate five minutes after each addition of Ca^2+^. Emission fluorescence was measured with an LS 55 Fluorescence Spectrometer (PerkinElmer). Excitation wavelength was 282 nm, and emission wavelength was 331 nm; 5 nm slit width was used for both excitation and emission.

A minimum of three FCSQ preparations was used for each experimental condition, and assays using each of the protein preparations were run in triplicate. Estimates for dissociation constant (K_d_) were calculated using the following equation [34]:
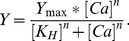
(1)where Y represents fluorescence, K_H_ represents the Hill constant, n represents the index of cooperativity or Hill coefficient, and [Ca] represents the calcium concentration. The Hill constant indicates the concentration of ligand that accounts for ½ the maximal response, which in the case of fluorescence assays is the concentration of calcium that results in ½ the maximal fluorescence. The K_d_ values were calculated as K_d_ = [K_H_]^n^. Standard error was calculated from the averages obtained for each FCSQ protein preparation. One-way ANOVA, followed by Tukey HSD multiple pairwise comparisons with significance set at 5% was used to determine significant differences in dissociation constants (K_d_) and Hill coefficients (n).

### Molecular Modeling

Sequences were aligned using ClustalW version 2.0 housed on the EBI server [29]. A homology model of FCSQ was built using the crystal structure of CSQ2 from dog (*Canis lupus familiaris*), DCSQ2 (PDB ID: 1SJI) as a template [4]. The first 20 residues of the coding sequence were identified as the SR targeting signal sequence and were removed prior to modeling. The C-terminal tail was trimmed to match the primary structure of DCSQ2.

The model was built using SwissModel [35]. The first two N-terminal residues and the C-terminal tail of FCSQ were truncated to match the template structure, producing a monomer with 350 residues and dimer with 700 residues (chain A residues 1–350 and chain B residues 351–700, equivalently 1′-350’). Model quality was validated using SwissModel built-in evaluation procedures. Visual Molecular Dynamics (VMD) [36] and Swiss-PDB Viewer [37] were used to visualize the molecules. A molecular model of FCSQ front-to-front dimer was made using PyMol [38].

The VMD Autopsf plugin was used to add missing hydrogen atoms and to create atom connectivity files (psf files) with the Charmm force field topology file top_all22_prot.inp [39]. The Salt Bridge plugin of VMD was used to calculate the distance between oxygen and nitrogen atoms in the protein and to determine which residues are involved in salt bridging based on a 3.2 Å distance constraint. Angles between participating oxygen and nitrogen atoms were calculated manually in VMD. An explicit TIP3P water box with 10 Å padding was created for each system, which was ionized with K^+^ ions to neutralize charge [40].

Electrostatic interaction energies, U(r), in solvated systems are estimated with Coulomb’s law screened by an effective dielectric constant:
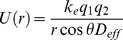
(2)where k_e_ is the Coulomb constant 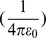, q is the charge, r is the distance between the basic nitrogen (donor) and the acidic oxygen closest to the donor (acceptor), θ is the angle between N-H••O, and D_eff_ is the effective dielectric constant. Indeed, the net contribution of a salt bridge interaction to protein folding free energy can only be obtained through computation [41].

A linear distance-dependent dielectric (DDD) [42] was chosen to estimate D_eff_:
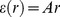
(3)where ε(r) is the functional form of D_eff_ in Equation 2, A is 2, and r is the distance between donor and acceptor. The constant, A, describes the level of solvent screening effect and is based on the level of exposure of the interaction to solvent. Since the interactions are buried in the dimer interface a value of 2 was chosen for A, indicating minor solvent exposure.

## Results and Discussion

### *F. heteroclitus* CSQ Primary Structure

**Figure 1:**
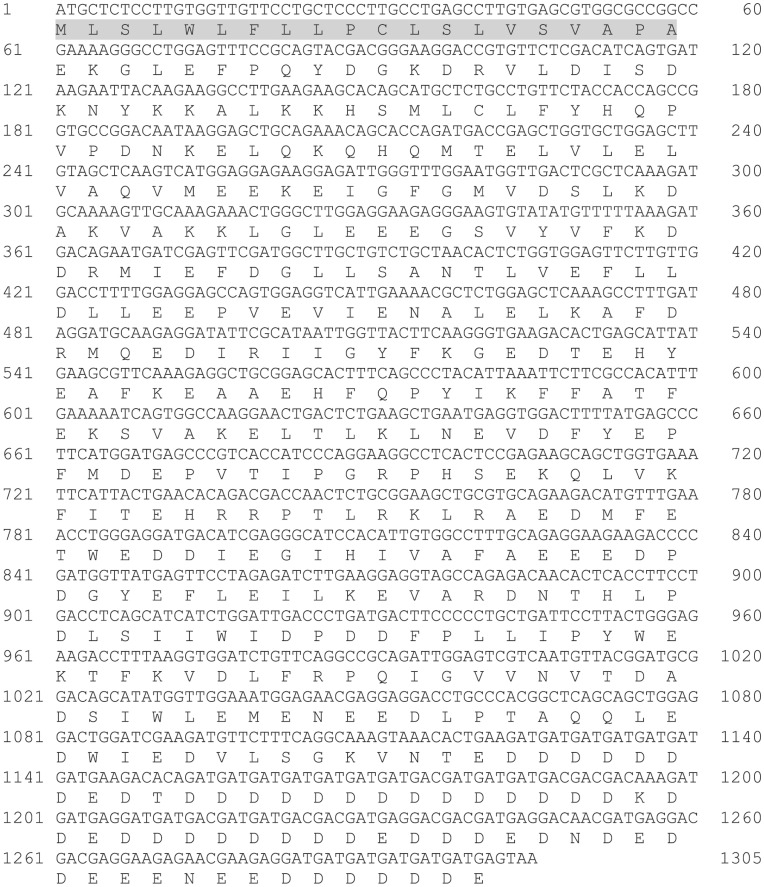
Nucleotide and deduced amino acid sequence of the coding region of FCSQ cDNA. Sequences were reconstructed from overlapping sequences of PCR amplified fragments. Primer sequences were removed prior to alignment. The putative SR targeting sequence is shaded.

We isolated and determined the full-length coding sequence of a CSQ isoform from the southern population of *F. heteroclitus* (FCSQ; NCBI Accession Number: HQ615687) with an open-reading frame of 1,305 bases (Figure 1). The first 20 amino acids, the SR targeting sequence, are cleaved after import into the SR [43]. The mature FCSQ deduced amino acid sequence is predicted to be 414 amino acids with a calculated molecular weight of 48,474 Da. Similar to other vertebrate CSQs, FCSQ has a high proportion of acidic residues with 137 of the 414 amino acid residues (33%) being aspartic acid and glutamic acid. These acidic amino acid residues are responsible for forming clusters of 2–3 amino acids that coordinate Ca^2+^, thus playing a critical role in the Ca^2+^-binding capacity of CSQ [44].

**Figure 2:**
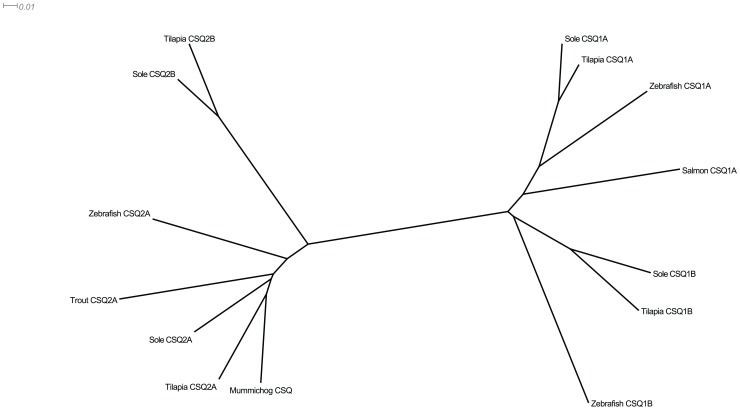
Neighbor-joining tree displaying relationships between CSQ orthologs and paralogs. Teleost fishes express multiple isoforms of CSQ in a variety of tissues. FCSQ (mummichog CSQ) aligns most closely with CSQ2A isoforms.

A phylogenetic analysis of FCSQ with other fish CSQ sequences shows FCSQ to be most similar to the oxidative skeletal muscle and cardiac isoform, CSQ2A (Figure 2). Primary sequence comparisons between FCSQ and other teleost CSQs show 56–60% identity (78–80% similarity) and 71–88% identity (88–97% similarity) for CSQ1 and CSQ2 isoforms, respectively. FCSQ cDNA was amplified from *F. heteroclitus* skeletal muscle cDNA using degenerate primers that were based on an alignment of CSQ1 and CSQ2 isoforms from *D. rerio*, *F. heteroclitus*, and *T. rubripes*, as described above. Due to the arrangement of oxidative and glycolytic fibers in teleosts, we cannot discount the presence of oxidative fibers in muscle samples that were used to isolate total RNA, which was subsequently used in RT-PCR and CSQ amplification. Therefore, based on sequence analysis, it is likely that the PCR reactions using degenerate primers amplified a CSQ isoform expressed in oxidative skeletal muscles, specifically, CSQ2A.

Recent research on CSQ expression in poikilothermic fishes has found that there are more than two isoforms of CSQ. The naming of CSQ isoforms in fishes seems to be based more on similarity to either mammalian CSQ1 or CSQ2 rather than tissue specific expression. Korajoki and Vornanen [45] identified multiple transcripts of CSQ2 in cardiac muscle of rainbow trout (*Oncorhynchus mykiss*) that are products of a single gene and not paralogous genes. In addition, Infante et al. [26] isolated two isoforms each of CSQ1 and CSQ2 from ESTs and cDNA libraries from the Senegalese sole (*Solea senegalensis*) that were not tissue specific. They also found that all four isoforms were expressed in ten different tissues [26]. An area of future research is to identify isoforms and expression patterns of CSQ in *F. heteroclitus* skeletal and cardiac muscles and to characterize the functions of these isoforms in this eurythermal teleost.

### Functional Characterization

**Figure 3:**
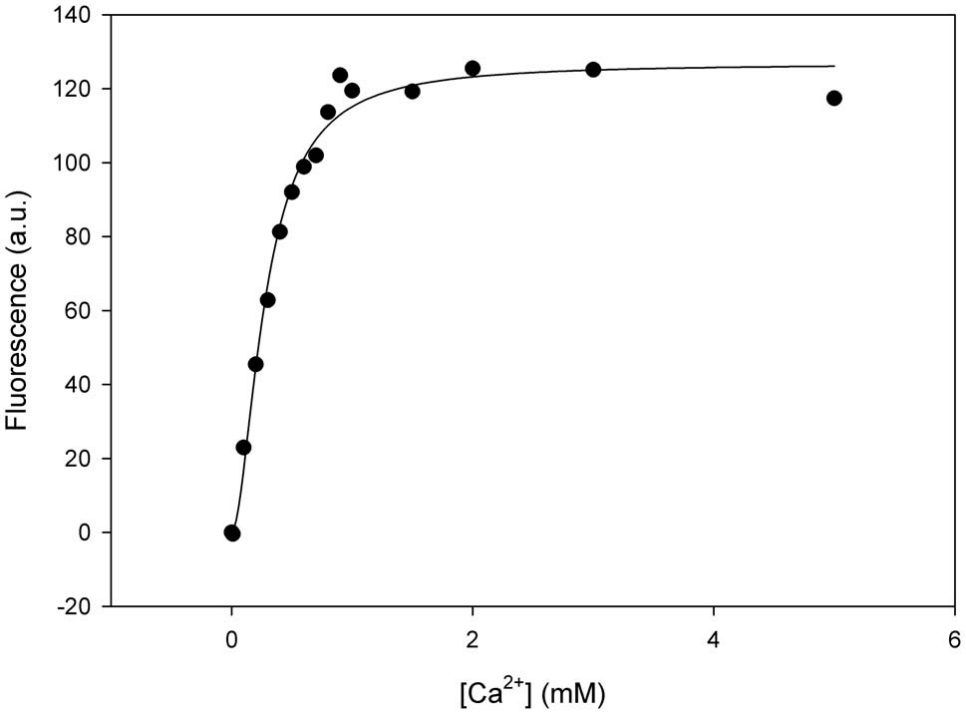
Representative fluorescence data of Ca^2+^-binding by FCSQ. Fitting of this data yields a K_d_ value of 102 µM and a Hill coefficient of 1.8.

This study is the first to investigate the sensitivity of FCSQ function to changes in temperature and pH. Dissociation constants of Ca^2+^ were determined using an assay that monitors intrinsic tryptophan fluorescence. Change in intrinsic tryptophan fluorescence in response to Ca^2+^ is interpreted as a conformational change of CSQ during Ca^2+^-binding and has been demonstrated in human cardiac CSQ [46]–[48] and dog cardiac CSQ [23]. Intrinsic fluorescence has also been used to determine the equilibrium binding constants [33] and dissociation kinetics of rabbit skeletal CSQ [20], [49]. This study is the first to apply this technique to CSQ from a teleost and to show that the intrinsic fluorescence of FCSQ is sensitive to Ca^2+^ (Figure 3).

**Figure 4:**
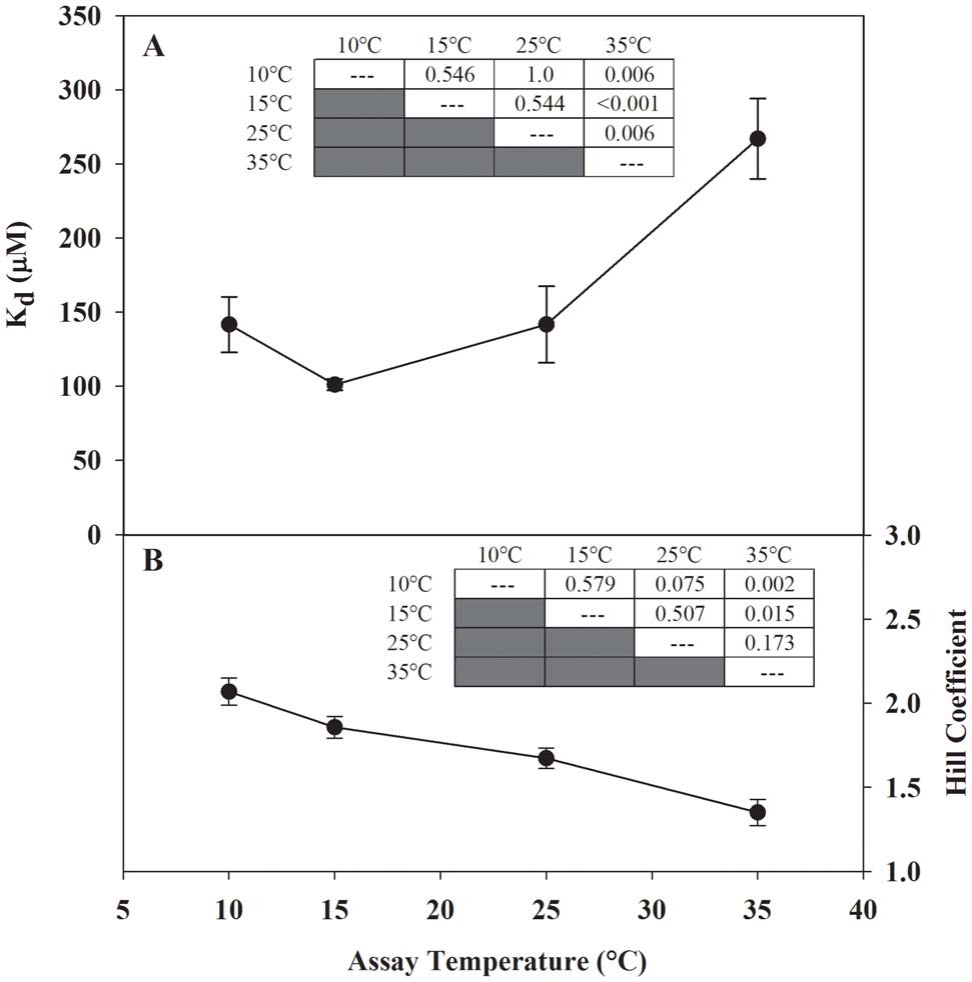
Effect of temperature on the apparent dissociation constant (Panel A) and Hill coefficient (Panel B) of Ca^2+^-binding by FCSQ. Each data point represents a minimum of three protein preparations run in triplicate. Significant differences were determined using one way ANOVA followed by Tukey HSD multiple pairwise comparisons with significance set at 5%. Inset tables show P values from all pairwise comparisons. Error bars represent S.E.M.

The FCSQ isoform examined in the current study is relatively insensitive to changes in temperature and pH. In the physiological temperature range for *F. heteroclitus*, 10°C to 25°C, there are no statistical differences in K_d_, but at 35°C the K_d_ is significantly different (higher) than the K_d_ values at the lower temperatures (Figure 4A). In laboratory acclimated animals a heat shock response is elicited at 35°C [50], which is at the high end of the thermal range of *F. heteroclitus*. The increase in K_d_ at 35°C, which correlates with the heat shock response, may be a result of partial unfolding or increased flexibility within regions of the protein that leads to a reduced affinity for Ca^2+^, and thus decreased Ca^2+^-binding ability at higher temperatures.

Interestingly, the longjaw mudsucker, *Gillichthys mirabilis*, which is distantly related to *F. heteroclitus*, has a similar eurythermal lifestyle and latitudinal distribution, but on the Pacific Coast. The apparent K_m_ vs. temperature curves for the eurytolerant pyruvate kinase (PK) isoform from *G. mirabilis* binding to its substrate, phosphoenolpyruvate, show a trend that is qualitatively similar to the K_d_ vs. temperature curve for FCSQ Ca^2+^-binding. In the physiological range of temperatures of *G. mirabilis* (10°C–30°C) the K_m_ of PK shows very little thermal sensitivity, but outside the range K_m_ increases rapidly [27], [51]. These convergent phenotypes from *G. mirabilis* PK and *F. heteroclitus* CSQ suggest that thermal insensitivity may be an adaptive response utilized in proteins from organisms that experience significant and rapid environmental variability.

The change in Hill coefficient (n) in response to temperature shows a similar trend to the K_d_ measurements (Figure 4B). The Hill coefficients determined at 10°C and 35°C are significantly different. There are no significant differences among Hill coefficients determined from 10°C to 25°C. There is, however, a qualitative decreasing trend in Hill coefficient values with increasing temperature. A similar trend has been reported for an oligomeric alcohol dehydrogenase from the archaeon *Sulfolobus solfataricus* [52]. The Hill coefficient is a measure of the sigmoidicity of the binding curve, which is a reflection of the level of cooperativity of the binding mechanism [34]. An n value of 1 indicates a non-cooperative binding mechanism, while n <1 indicates negative cooperativity, and n >1 indicates positive cooperativity. The cooperative mechanism of CSQ Ca^2+^-binding can be easily described using the scheme outlined in the Introduction. As [Ca^2+^] increases, CSQ folds and forms front-to-front and back-to-back dimers where the majority of Ca^2+^ is coordinated [22]. As the number of dimer interfaces increases, Ca^2+^-binding capacity of CSQ increases. Making the assumptions that the Hill coefficient reports on cooperativity, and cooperativity is mediated by polymerization, the decrease in Hill coefficient in response to increased temperature suggests that higher temperature inhibits FCSQ polymerization.

**Figure 5:**
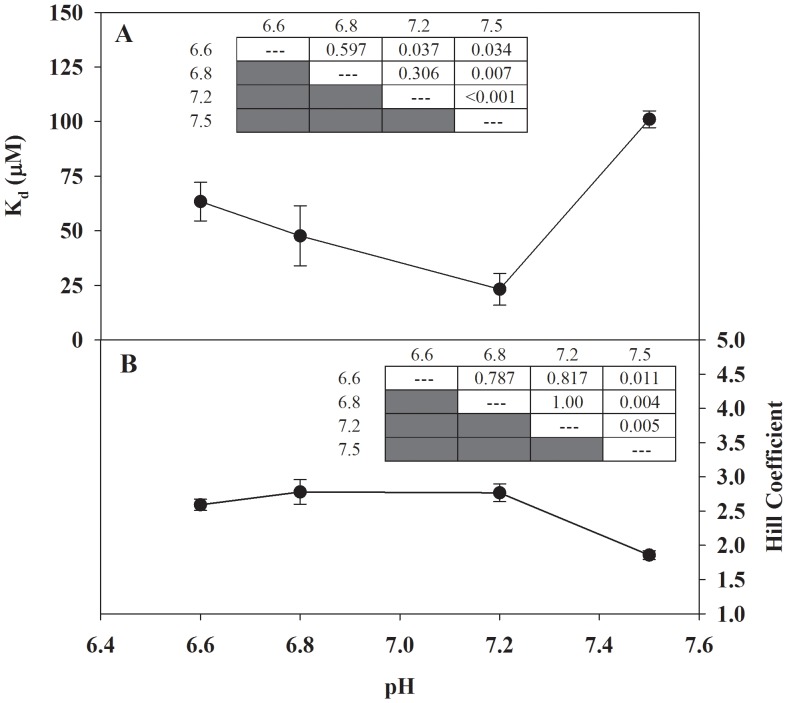
Effect of pH on the apparent dissociation constant (Panel A) and Hill coefficient (Panel B) of Ca^2+^-binding by FCSQ. Each data point represents a minimum of three protein preparations run in triplicate. Significant differences were determined using one way ANOVA followed by Tukey HSD multiple pairwise comparisons with significance set at 5%. Inset tables show P values from all pairwise comparisons. Error bars represent S.E.M.

As intracellular temperature changes in a poikilothermic vertebrate, the intracellular pH also changes [53]–[55]; generally, for every 1°C increase in temperature, pH decreases by approximately 0.017 units [53]. Both temperature and pH have been shown to affect *F. heteroclitus* protein function [56], and the pH values used in our K_d_ vs. pH experiments are physiologically relevant to changes in pH that occur in this teleost. There is no significant difference in K_d_ values between pH 7.2 and 6.8, or between 6.8 and 6.6, but the K_d_ value calculated at pH 7.5 is statistically different (higher) then the K_d_ values calculated at lower pH (i.e. 6.6, 6.8, and 7.2; Figure 5A). The trend in Hill coefficients mirrors that for K_d_ values (Figure 5B). Overall, FCSQ function appears to be relatively insensitive to pH changes. This relative insensitivity of FCSQ function to pH variation contrasts with the function of rabbit skeletal CSQ [33]. While the Hill constant (K_H_) of rabbit CSQ remained relatively constant (∼300 µM), the Hill coefficient (n) decreased from 4.4 at pH 7.5 to ∼1 at pH 6.0 [33], which results in an inverse relationship between pH and K_d_. The changes in Ca^2+^-binding ability and cooperativity of rabbit CSQ as a function of pH suggest that the contrasting functional profile of FCSQ may be an adaptive response of *F. heteroclitus* to the variable intertidal environment.

### Structural Characterization

To investigate the structural basis of polymerization in FCSQ we characterized the electrostatic interactions at the front-to-front dimeric interface. The crystal structure of dog cardiac CSQ (DCSQ2) was used as the template for a homology model of FCSQ. The sequence identity between the target (FCSQ) and template (DCSQ2) sequences is 68.3%, well above the “twilight zone” threshold for homology modeling [57]. At this level of sequence identity SwissModel produces reliable models with resolutions similar to experimentally determined structures [58]. The root mean square deviation (RMSD), a measure of difference between two macromolecular structures, is 1.10 Å between the template and target structures, which is within the resolution of the template structure, 2.40 Å.

**Figure 6:**
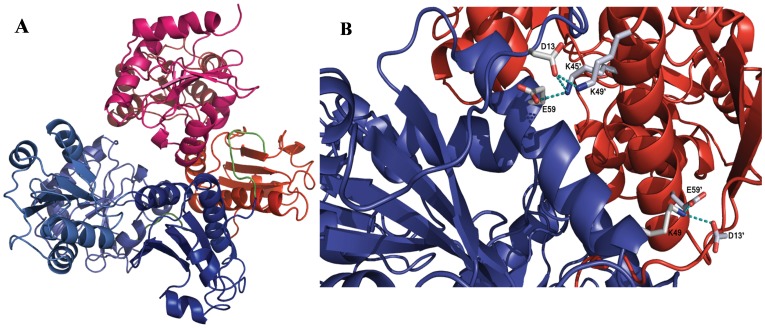
Model of FCSQ front-to-front dimer. Panel A shows Chain A (blue) and Chain B (red) interacting to form an electronegative pocket where Ca^2+^ is coordinated by pairs of negatively charged residues. During front-to-front dimerization Domain I from each monomer interacts at the N-terminal end of the molecules. The N-terminal tails that participate in dimerization are shown in green. Panel B shows the salt bridge network that stabilizes the interactions between Domain I of Chains A and B. Participating side chains are shown in CPK coloring. Chain coloring as in panel A. Electrostatic interactions (salt bridges) are shown as dashed, cyan lines.

Based on the homology model of FCSQ, monomers of FCSQ will form front-to-front dimers (Figure 6A). Table 2 shows the residues involved in intermonomeric salt bridges and distances found in this analysis in both the template and target structures. Interestingly, in this model and in the crystal structure of DCSQ2 the residues are symmetrical, but the interactions are not. While the salt bridge between D13–K45’ (45′ indicates the opposing monomer) is present on one side of the dimer (Figure 6B), the reverse interaction, D13’-K45, is not fully formed. Similarly, the salt bridge between E300–K68’ is fully formed, while E300’-K68 is not present. Although not a symmetric interaction, this pair of salt bridges seems to be cooperative. When D13–K45’ and E300–K68’ are fully formed, D13’-K45 and E300’-K68 are not, causing one fully formed salt bridge interaction point between Domain I of monomer A and Domain I of monomer B. Most likely during a dynamic breathing mode of the protein, the intact salt bridge pair will swap back and forth while the more stable interactions between residues 13 and 49, and 49 and 59 of both monomers remain intact.

**Table 2:** Intermonomeric salt bridges of FCSQ and DCSQ2.

	FCSQ				Dog CSQ2			
Salt Bridge	Distance (Å)	U(r) (kcal/mol)	ε(r) = D_eff_	U(ε(r)) (kcal/mol)	Distance (Å)	U(r) (kcal/mol)	ε(r) = D_ef_	U(ε(r)) (kcal/mol)
D13–K45’	3.01	18.79	6.0	3.12	2.95	21.47	5.9	3.64
D13–K49’	2.86	23.61	5.7	4.13	2.91	23.85	5.8	4.10
D13’–K49	2.90	15.43	5.8	2.66	2.89	15.84	5.8	2.74
E300–K68’	3.11	22.94	6.2	3.69	2.95	22.54	5.9	3.82
E59–K49’	3.08	22.29	6.2	3.62	3.02	22.67	6.0	3.75
E59’-K49	2.74	26.76	5.5	4.88	2.83	26.47	5.7	4.68

Distance is measured from the center of mass of the nitrogen basic residue (donor) and the center of mass of the oxygen in the acidic residue (acceptor). U(r) is the interaction energy with no solvent screening effect. D_eff_ is as described in the methods using a value of A = 2. U(ε(r)) is the interaction energy calculated using D_eff_ that incorporates solvent screening.

Confirmation of the importance of the residues involved in these salt bridges is provided by the work of Bal et al. [28]. Using rat cardiac CSQ they found two N-terminal charge clusters that are critical for CSQ polymerization and function. They found that residues D32, K64, and K68 are involved in an intermonomeric salt bridge network that stabilizes front-to-front dimers [28]. These residues correspond to residues E13, K45, and K49 in our model of FCSQ. That these residues are conserved from fish to mammal is strong evidence that they are important for CSQ function across isoforms and species. This network acts as an important tether holding front-to-front dimers together (Figure 6A, B) [28]. In conjunction with the N-terminal arm, this tether helps position opposing CSQ monomers to allow pairs of negatively charged residues to bind Ca^2+^ molecules. Removal of any of these salt bridges should alter Ca^2+^-binding affinity and capacity, most likely negatively. Indeed, Bal et al. [28] found that alteration of the corresponding residues in rat cardiac CSQ diminished Ca^2+^-binding ability.

The strength of salt bridge interactions depends on pH, angle of the interaction, and distance between interacting residues, as well as the indirect effects of the solvent [59]. The interaction energies (U(r)) are given for both FCSQ and DCSQ2 using a D_eff_ of 1 (Table 2), which is essentially using Coulomb’s law to measure the interaction of the residues in vacuum and thus yields high values for the salt bridge energy. U(ε(r)), in contrast, incorporates solvent screening effects by using a constant, A, as described by Mehler and Eichele [42]. This functional form of D_eff_ is based on empirical data and provides a method of accounting for the shielding effects of the solvent when calculating salt bridge interaction energies. An A of 1 indicates an interaction buried in the protein interior with very little screening effects of the solvent; an A of 2 indicates a moderately solvent exposed interaction, and an A of 4 indicates a surface interaction that is highly exposed to solvent. While the residues involved in the tethering site are separated from solvent by only about 3 Å or more, the specific salt bridging atoms are protected from solvent by non-salt bridging atoms, giving at least one van der Waals layer of protection from solvent effects, and therefore are considered buried with moderate solvent exposure. The most solvent exposed residues are K68 (K68’) and E300 (E300’), but again, the specific salt bridging atoms are protected, in this case from other protein residues. Since all of the salt bridge interactions identified here are buried in the dimer interface, an A of 2 was chosen. While this model is an approximation method, the interaction energies calculated in this study correspond to values derived by experimental methods and other computational methods, and as such support our methodologies employed in this research [59], [60].

### FCSQ Function as an Adaptation

Temperature fluctuations can affect all aspects of protein structure and function including ligand binding and oligomerization [3], two processes that are critical to CSQ function. The typical pattern of amino acid substitutions for thermal adaptation found in enzymatic systems leads to small changes in structure away from the active sites. These changes appear to modulate function through changes in conformational flexibility [3], [61], [62], [63]. Recently, this paradigm of thermal adaptation elucidated for enzymes was extended to the non-catalytic protein parvalbumin (PV), which binds Ca^2+^ in the nanomolar range [64]–[66]. Parvalbumin is a member of the canonical, EF-hand family of Ca^2+^- binding proteins. EF-hand proteins have a highly conserved helix-loop-helix motif that coordinates Ca^2+^ with high specificity and high affinity [67]. In addition, a similar mode of thermal adaptation has been demonstrated in troponin C (TnC), which is also an EF-hand Ca^2+^-binding protein [68]–[71]. However, it has yet to be established if the same underlying principles governing thermal adaptation of PV and enzymes apply to the non-canonical CSQ. In contrast to PV and TnC, CSQ binds Ca^2+^ in a non-specific manner with clusters of acidic residues transiently coordinating Ca^2+^ ions in the electronegative pockets formed between dimers [22].

Previously, thermal compensation has been demonstrated in proteins for which polymerization is critical to function (e.g. actin and tubulin). Actins from a variety of vertebrates adapted to different temperatures show thermal stability and polymerization thermodynamics that correlate with physiological temperature [72]. While a specific primary structure-based mechanism was not determined, it is likely that the differences found among different actins are due to amino acid substitutions that lead to differences in structure, which conserve polymerization ability at physiological temperature. A specific structural mechanism has been found for polymerization of microtubules from cold-adapted Antarctic fish. Longitudinal interfaces of microtubule monomers are highly conserved, while adaptive substitutions occur in the monomer core and on the lateral interfaces. These substitutions affect a small number of non-covalent interactions that conserve the polymerization ability of the microtubules at physiological temperature [73].

The functional results reported here suggest that the in vitro function of FCSQ correlates with the natural history of *F. heteroclitus*. The residues involved in polymerization and Ca^2+^- coordination in CSQ are highly conserved suggesting substitutions that modulate function in response to environmental, and thus physiological, temperature occur elsewhere in the molecule. Further comparative studies of FCSQ and CSQ isoforms from fishes native to other thermal habitats will be needed to determine if the paradigm of thermal adaptation in enzymes and EF-hand proteins applies to CSQ.
